# Night blood pressure variability, brain atrophy, and cognitive decline

**DOI:** 10.3389/fneur.2022.963648

**Published:** 2022-09-01

**Authors:** Ji Hee Yu, Regina E. Y. Kim, So Young Park, Da Young Lee, Hyun Joo Cho, Nam Hoon Kim, Hye Jin Yoo, Ji A Seo, Seong Hwan Kim, Sin Gon Kim, Kyung Mook Choi, Sei Hyun Baik, Chol Shin, Nan Hee Kim

**Affiliations:** ^1^Division of Endocrinology and Metabolism, Department of Internal Medicine, Korea University College of Medicine, Seoul, South Korea; ^2^Institute of Human Genomic Study, Korea University Ansan Hospital, Korea University College of Medicine, Ansan, South Korea; ^3^Department of Psychiatry, University of Iowa, Iowa City, IA, United States; ^4^Division of Cardiology, Department of Internal Medicine, Korea University College of Medicine, Seoul, South Korea

**Keywords:** night blood pressure, variability, gray matter, brain atrophy, cognition

## Abstract

**Background:**

Although blood pressure variability (BPV) has emerged as a novel risk factor for Alzheimer's disease, few studies have examined the effects of night BPV on brain structure and function. This study investigated the association of night BPV with brain atrophy and cognitive function changes.

**Methods:**

The analysis included 1,398 participants with valid ambulatory blood pressure (BP) monitoring at baseline and both baseline and 4-year follow-up brain magnetic resonance images who were recruited from the Korean Genome and Epidemiology Study. Participants underwent a comprehensive neuropsychological test battery. BPV was derived from ambulatory BP monitoring and calculated as a standard deviation (SD) of 24-h and daytime and nighttime BP.

**Results:**

During the median follow-up of 4.3 years, increased SD of night systolic or diastolic BP was an indicator of total brain volume reduction, while daytime BPV or night average BP was not associated with total brain volume changes. High SD of night systolic BP was associated with reduced gray matter (GM) volume, independent of average night BP, and use of antihypertensive drugs. It also was associated with a reduction of temporal GM volume, mostly driven by atrophy in the left entorhinal cortex and the right fusiform gyrus. In cognitive performance, high variability of night systolic BP was associated with a decrease in visual delayed recall memory and verbal fluency for the category.

**Conclusion:**

Increased night BPV, rather than night mean BP, was associated with reduced brain volume and cognitive decline. High night BPV could be an independent predictor for rapid brain aging in a middle-aged population.

## Introduction

High blood pressure (BP) has been shown to be associated with brain atrophy and cognitive dysfunctions (1–3). Nocturnal hypertension showed significant associations with brain volume and cognitive impairment (1, 4, 5). In addition to the effect of BP level on brain function, the importance of dynamic changes in BP level in cerebrovascular disease has been supported by several observations. Past studies have focused on the effect of circadian BP variation, such as nocturnal BP dipping or non-dipping, and both patterns had adverse effects on brain health. Non-dippers are defined as night BP decrease <10% of the day-time level and showed more frequent silent cerebral infarction than dippers, with a night BP decrease >10% (6). In contrast, the very large nocturnal BP decrease of an extreme dipper could induce cerebral vascular insufficiency (7). Since the advent of 24-h non-invasive ambulatory BP monitoring, most studies have evaluated the association between short-term BP variability (BPV) and cerebral outcomes. High BPV was associated with an increased risk of cerebral small vessel disease, stroke, dementia, and cognitive decline (8–13). Evidence has shown that greater BPV leads to diffuse atherosclerotic progression represented by increased left ventricular mass index or carotid-intima media thickness value (14, 15).

Despite these observations, the relationship between increased nighttime BPV and brain volume atrophy or cognitive function remains poorly understood. Previous studies are limited by small sample sizes (16) or cross-sectional associations with inconsistent results (17–19). Moreover, it is not clear which regional area of the brain is most associated with high night BPV and how it relates to changes in cognitive functions.

In this study, we investigated whether increased night BPV is associated with brain volume atrophy and cognitive decline in a middle-aged population. We analyzed longitudinal associations between night BPV at baseline and changes in brain volume and cognitive function across 4 years.

## Materials and methods

### Subjects

The study subjects were from the Ansan cohort of the Korean Genome Epidemiology Study (KoGES), an ongoing population-based cohort study that began in 2001. The demographics, medical illness, and medications of KoGES participants have been biennially evaluated. During the 6th and 7th examinations (2011–2014), baseline brain magnetic resonance imaging (MRI) scans and cognitive function tests were acquired. Follow-up brain MRI scans and cognitive function tests were conducted during the 8th and 9th examinations (2015–2018). Further details are described elsewhere (20).

**Figure 1:**
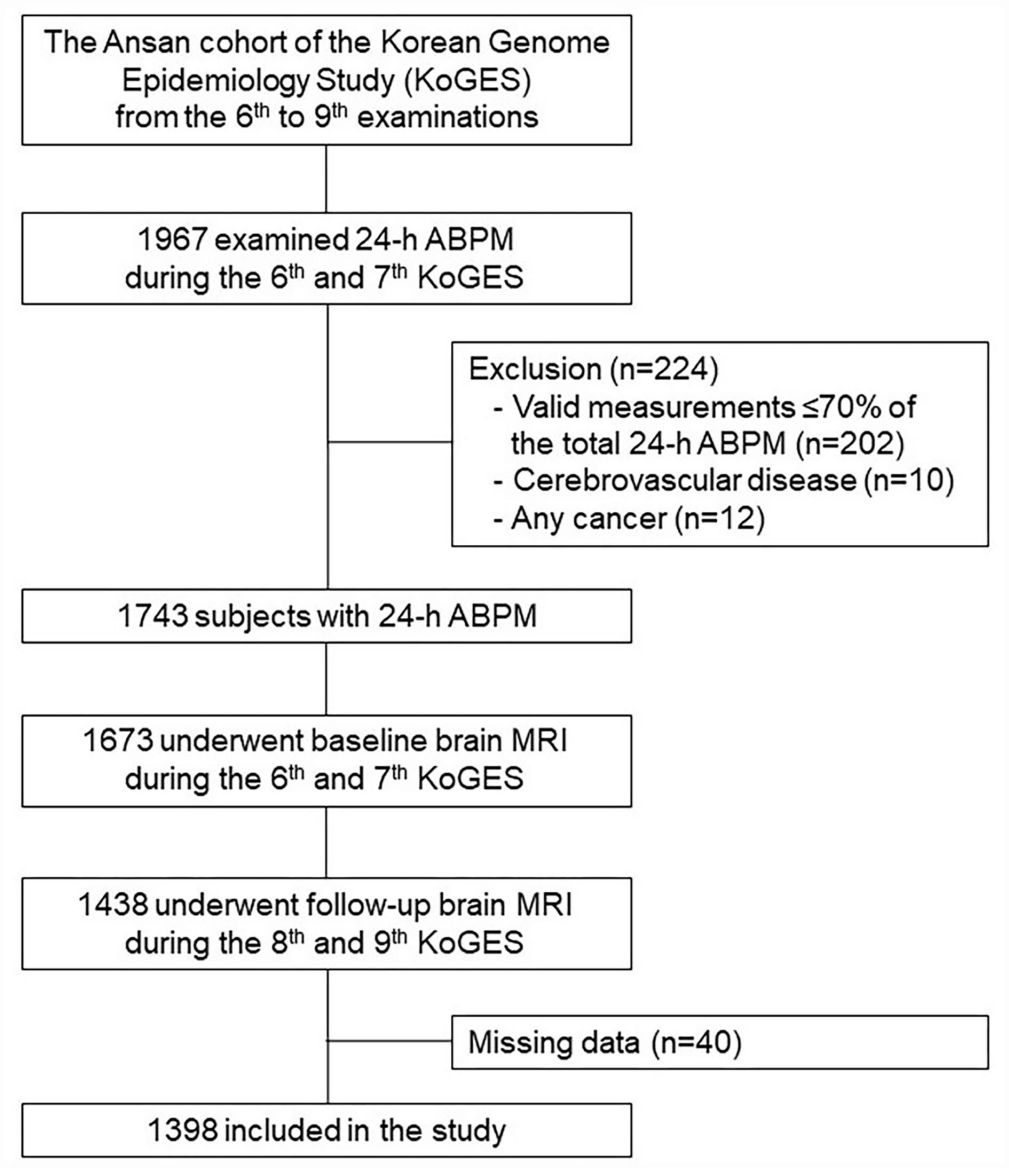
A flowchart of the selection process. ABPM, ambulatory blood pressure measurement; MRI, magnetic resonance imaging.

This study included 1,967 subjects who examined 24-h ABPM during the baseline period (2011–2014) (Figure 1). We excluded 224 subjects with the following conditions: (1) valid 24-h ABPM ≤ 70% of the readings of total 24-h BP measurements (*n* = 202); (2) cerebrovascular disease (*n* = 10); and (3) any cancer (*n* = 12). Among the remaining 1,743 subjects, 1,438 individuals underwent both baseline and follow-up brain MRI scans. None of them had a history of dementia or any neuropsychiatric disorders. After the exclusion of subjects with missing data (*n* = 40), 1,398 participants were finally included in this study. This study was performed according to the principles of the Declaration of Helsinki of the World Medical Association and was approved by the Institutional Review Board of Korea University Ansan Hospital.

### Assessments

#### Demographic, anthropometric, and laboratory measurements

All participants responded to an interviewer-administered questionnaire and underwent physical examinations. Lifestyle characteristics, such as smoking status and alcohol consumption, were categorized as never, former, and current. Regular exercise was defined as at least three times a week for 30 min per session during the previous month. Education level was categorized into primary, secondary, and college/university levels. Height was measured to the nearest 0.1 cm using a fixed wall-scale measuring device. Weight was measured to the nearest 0.1 kg using an electronic scale that was calibrated before each measurement. Body mass index (BMI) was calculated as weight in kilograms divided by height in meters squared. Blood was drawn for biochemical analysis after an overnight fast.

#### 24 h ABPM

A Mobil-O-Graph NGversion20, which is a non-invasive oscillometric device, and its hypertension management software (I.E.M. GmbH, Stolberg, Germany) were used for ABPM. A trained researcher informed the participant during a clinic visit how to use the home 24-h ABPM device. A BP cuff was placed on the upper region of the non-dominant arm, and BP was recorded automatically every 30 min (0600–2,300 h) or every hour (2,300–0600 h). The participant was asked to record the time of waking and sleeping over a 24-h period. Daytime and nighttime for each of the participants were ascertained based on the awake and asleep times.

#### Brain MRI

All 3D T1 MRI scans were acquired using a GE Signa HDxt 1·5 T MRI scanner with an 8-channel head coil. The detailed MRI protocols are described in a previous study (20). Brain MRI images were processed through a well-established fully automated procedure, the BRAINS AutoWorkup in the BRAINSTOOLs package (21, 22). The MRI processing starts with spatial normalization using landmark detection (23), bias-field correction with tissue classification (22), and finally segmentation using ANTs Joint Fusion (24). Two hundred fifteen independent brain subcompartments were automatically delineated, and the volumes were measured. The high reliability of the longitudinal measurement of two-time point MRI using the BRAINS AutoWorkup has been previously described (20). The sub-compartments were merged into three tissue classes: GM, WM, and cerebrospinal fluid. All the volume measurements were extracted from an individual's original anatomical space. GM and WM volumes were summed to obtain total brain volume.

#### Neuropsychological tests

KoGES participants were administered the neuropsychological assessment battery described below during the regular examination cycle as part of the baseline measurement of the aging study: (1) story recall test, immediate and delayed recall, and recognition; (2) visual reproduction, immediate and delayed recall, and recognition; (3) verbal fluency; (4) trail making tests; (5) Digit Symbol-coding, incidental learning, and free recall; and (6) Korean-Color Word Stroop Test, word reading, and color reading. Standard administration protocols were used for each testing session, and the tests were conducted by well-trained and experienced psychological examiners. Further details are described elsewhere (25).

#### Definitions of diabetes mellitus (DM), hypertension, heart disease, and obesity

DM was diagnosed as fasting plasma glucose ≥7.0 mmol/L, 2-h plasma glucose ≥11.1 mmol/L after a 75 g oral glucose tolerance test or use of anti-diabetic medication (26). Hypertension was diagnosed as systolic BP (SBP) or diastolic BP (DBP) equal to or higher than 140 or 90 mmHg, respectively, or use of antihypertensive medications. Participants with a documented history of myocardial infarction, angina, or congestive heart failure were considered to have heart disease. Obesity was defined as BMI ≥25 kg/m^2^.

#### Sleep duration and snoring measurements

All participants were asked to answer sleep-related questions based on the average sleep pattern during the past month. Sleep duration was determined as the answer to, “How many hours did you usually sleep per day during the last month?” Participants were asked if they had ever been reported to snore. Snoring status was confirmed by a bed partner or a family member who lived with the participant for more than 1 year. Further details are described elsewhere (27).

### Statistical analysis

As measures of short-term reading-to-reading BPV, we used the SD over daytime and nighttime. Baseline characteristics are presented as number (%) or mean ± SD. The brain volume change was calculated by subtracting the baseline brain volume from the follow-up brain volume. Multivariate linear regression analyses were conducted to evaluate the effects of BPV on brain volume or cognitive function changes. The regression models included brain volume changes as the dependent variable; ICV, age, sex, smoking, alcohol, exercise, education, an average of daytime and nighttime BP, antihypertensive medications, DM, heart disease, baseline brain volume, and time intervals between baseline and follow-up MRI scans were included as the independent variables. For analysis of associations with night systolic BPV, the average SBP during the day and that during the night were included in the regression models; for analysis of night diastolic BPV, the average DBP values during both days and night were included. The association of night BPV with brain regional volume changes was further analyzed in sub-compartments of WM and temporal GM. The cognitive function change was calculated by subtracting the baseline cognitive scores from the follow-up scores. The regression models included cognitive function change as the dependent variable, and baseline cognitive scores and time between cognitive tests were entered as variables along with those mentioned above. Sobel's tests were performed to examine whether GM volume atrophy mediated any associations between night systolic BPV and decline in cognition. Statistical significance for non-normally distributed cognitive variables was estimated after logarithmic transformation. A *P*-value <0.05 was considered statistically significant. Statistical analyses were performed using SAS version 9.1 for Windows (SAS Institute Inc., Cary, NC, USA).

## Results

### Subject characteristics

The descriptive and clinical characteristics of the study population at baseline are presented in Table 1. The mean age of all participants was 59.7 ± 6.7 years (range, 49–79), and 46.0% of the individuals were men. The mean BMI was 24.6 ± 2.9 kg/m^2^, and 43.3% of the participants were obese. Approximately, 38.5% of patients had hypertension and 31.8% had diabetes mellitus at baseline. In addition, 13.1% of participants had an education level of elementary school or lower.

**Table 1:** Baseline characteristics of the study subjects.

***N* = 1,398**	
Age, years	59.7 ± 6.7
Age group	
<60 years	796 (56.9)
60–69 years	454 (32.5)
≥70 years	148 (10.6)
Sex, men	643 (46.0)
Current smoker	132 (9.4)
Current drinker	597 (42.7)
Regular exercise	527 (37.7)
Education, elementary or less	183 (13.1)
Primary	183 (13.1)
Secondary	912 (65.2)
College/university	303 (21.7)
BMI, kg/m^2^	24.6 ± 2.9
Day SBP, mmHg	122.6 ± 12.0
Day DBP, mmHg	80.7 ± 10.1
Night SBP, mmHg	112.3 ± 12.8
Night DBP, mmHg	72.1 ± 10.0
SD of day SBP	12.6 ± 4.0
SD of day DBP	9.6 ± 2.5
SD of night SBP	10.1 ± 4.5
SD of night DBP	8.6 ± 3.5
ICV, mL	1396.1 ± 137.1
DM	444 (31.8)
Hypertension	538 (38.5)
Antihypertensive medications	454 (32.5)
Heart disease	107 (7.7)
Obesity	605 (43.3)
Sleep duration, h	6.0 ± 1.2
Snoring	1,072 (77.2)

Data are presented as mean ± SD or number (%).

BMI, body mass index; DBP, diastolic blood pressure; DM, diabetes mellitus; ICV, intracranial volume; SBP, systolic blood pressure; SD, standard deviation.

### Associations between BPV and total brain volume changes

Table 2 presents the associations between the 24-h ambulatory BP indicators and total brain volume changes. The average period between baseline and follow-up brain MRI scans was 4.3 ± 0.5 years. The mean BP during daytime or nighttime was not associated with total brain volume changes. In relation to BPV, only SD of night BP was significantly associated with total brain volume changes. Higher SD of night SBP or DBP was significantly associated with greater total brain volume reduction after full adjustment (*P* = 0.020 for SD of night SBP, *P* = 0.015 for SD of night DBP; Table 2). Further adjustment for sleep duration, snoring, or BMI did not alter the significance of these associations. The SD of night SBP or DBP was positively associated with age, BMI, and an average of day and night BP in the multivariable regression analysis (Supplementary Table 1). Sleep parameters such as sleep duration or snoring were not associated with night BPV.

**Table 2:** Associations between 24-h BP indicators and total brain volume changes.

	**SBP**			**DBP**		
***N* = 1398**	**β Estimate**	**SE**	***p***	**β Estimate**	**SE**	***p***
Mean day	0.058	0.063	0.357	0.059	0.081	0.470
Mean night	0.0004	0.059	0.995	0.022	0.078	0.773
SD day	−0.055	0.158	0.728	−0.054	0.220	0.807
SD night	−0.278	0.119	**0.020**	−0.375	0.154	**0.015**

All models are adjusted for baseline intracranial volume, age, sex, smoking, alcohol, exercise, education, mean day BP, mean night BP, anti-hypertensive medications, DM, heart disease, time between MRI scans, and baseline brain volume. Brain volume changes were calculated by subtracting baseline brain volumes from follow-up brain volumes.

BP, blood pressure; DBP, diastolic blood pressure; DM, diabetes mellitus; MRI, magnetic resonance imaging; SBP, systolic blood pressure; SD, standard deviation. Bold values indicate *P* value < 0.05.

### Effects of night BPV on regional brain volume changes

**Figure 2:**
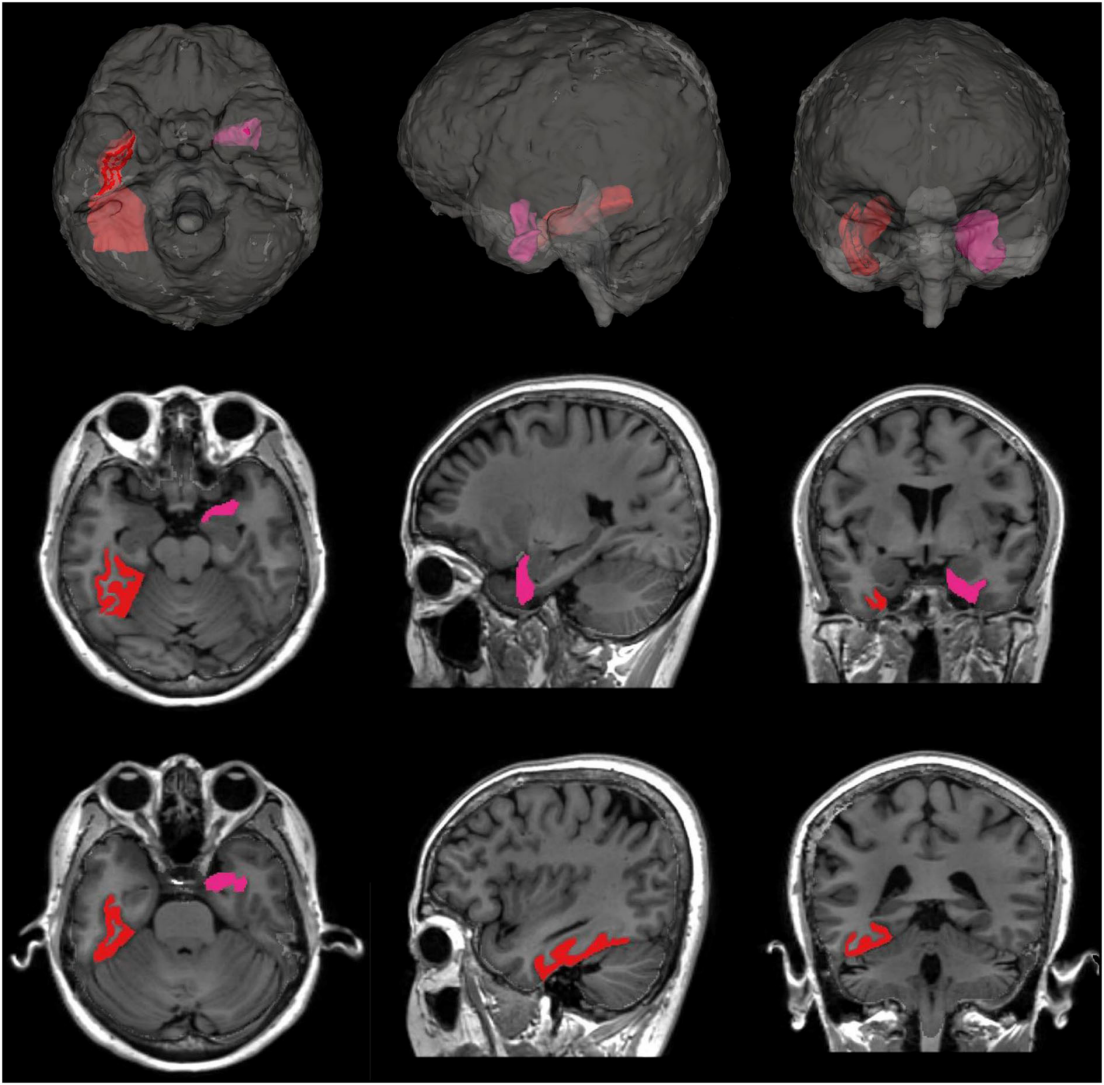
Subregions of temporal GM with significant atrophy associated with night systolic BPV. The left entorhinal cortex (marked in pink, *P* = 0.010) and the right fusiform gyrus (marked in red, *P* = 0.039) showed significantly greater atrophy associated with increased night systolic BPV.

We evaluated the effects of night BPV on regional brain volume changes, and the results are shown in Table 3. Increased SD of night SBP was associated with greater atrophy in GM volume (*P* = 0.033), particularly with decreased temporal GM (*P* = 0.021). The significance of this finding did not change even after further adjustment for sleep duration, snoring, or BMI. The relationship between night systolic BPV and temporal GM was mostly driven by atrophy of the left entorhinal cortex (*P* = 0.010) and right fusiform gyrus (*P* = 0.039) (Figure 2; Supplementary Table 2). Atrophy of the right fusiform gyrus was also associated with increased SD of night DBP (*P* = 0.018).

**Table 3:** Linear regression analyses between night BPV and regional brain volume changes.

	**SD of night SBP**			**SD of night DBP**		
**Δ ml (*n* = 1,398)**	**β Estimate**	**SE**	***p***	**β Estimate**	**SE**	***p***
Δ GM	−0.168	0.079	**0.033**	−0.159	0.102	0.121
Δ Frontal GM	−0.035	0.043	0.422	−0.039	0.056	0.485
Δ Parietal GM	−0.033	0.019	0.090	−0.021	0.025	0.411
Δ Temporal GM	−0.039	0.017	**0.021**	−0.018	0.022	0.407
Δ Occipital GM	−0.014	0.015	0.335	−0.015	0.019	0.442
Δ WM	−0.127	0.092	0.168	−0.234	0.119	**0.049**
Δ Frontal WM	−0.039	0.039	0.318	−0.096	0.050	0.055
Δ Parietal WM	−0.037	0.026	0.160	−0.060	0.034	0.073
Δ Temporal WM	−0.003	0.019	0.885	−0.013	0.025	0.592
Δ Occipital WM	−0.013	0.009	0.171	−0.015	0.012	0.225

All models were adjusted for baseline intracranial volume, age, sex, smoking, alcohol, exercise, education, mean day BP, mean night BP, anti-hypertensive medications, DM, heart disease, time between MRI scans, and baseline brain volume.

Regional brain volume changes were calculated by subtracting baseline regional brain volumes from follow-up volumes.

BP, blood pressure; DBP, diastolic blood pressure; DM, diabetes mellitus; GM, gray matter; MRI, magnetic resonance imaging; SBP, systolic blood pressure; SD, standard deviation; WM, white matter.

SD of night DBP showed a negative association with WM volume changes (*P* = 0.049; Table 3). Higher night diastolic BPV was associated with greater regional WM atrophy in the right precentral (*P* = 0.045), left paracentral (*P* = 0.025), right rostral middle frontal (*P* = 0.048), both superior frontal (*P* = 0.024 for left, 0.049 for right), left precuneus (*P* = 0.033), left transverse temporal (*P* = 0.027), both lingual (*P* = 0.016 for left, 0.034 for right), and right cuneus (*P* = 0.029) gyri (Supplementary Table 3). The volume changes of the hippocampus were not associated with night systolic or diastolic BPV (data not shown).

### Effects of night BPV on cognitive performance changes

Table 4 shows the associations between night systolic BPV and cognitive performance changes. High SD of night SBP was significantly associated with a greater decline in visual delayed recall memory (*P* = 0.028) and verbal fluency for category (*P* = 0.029) and with a slower decline in verbal recognition memory (*P* = 0.010) during the follow-up period. Further adjustment for sleep duration and snoring did not alter the significance of these associations. In contrast, night mean SBP or DBP was not associated with any cognitive functional changes (Supplementary Table 4). Night diastolic BPV was also not significantly associated with cognitive decline (data not shown).

**Table 4:** Linear regression analyses between night systolic BPV and cognitive performance changes.

	***n***	**Estimate**	**SE**	***p***
Story Recall Test-Immediate Recall	1,376	0.015	0.023	0.507
Story recall test-delayed recall	1,367	0.016	0.023	0.490
Story recall test-recognition^a^	1,379	0.003	0.001	**0.010**
Visual reproductions-immediate recall	1,384	−0.019	0.014	0.150
Visual reproductions-delayed recall	1,377	−0.032	0.015	**0.028**
Visual reproductions-recognition	1,380	−0.004	0.006	0.567
Verbal fluency-phonemic	1,347	0.013	0.045	0.768
Verbal fluency-category	1,347	−0.044	0.020	**0.029**
Digit symbol-coding	1,361	−0.022	0.042	0.598
Digit symbol-incidental learning^a^	1,343	0.002	0.004	0.660
Digit symbol-free recall	1,358	−0.003	0.008	0.727
Trails A-Time^a^	1,378	0.003	0.002	0.078
Stroop-color reading	1,333	0.127	0.137	0.352
Stroop-word reading	1,307	0.022	0.046	0.633

All models were adjusted for baseline intracranial volume, age, sex, smoking, alcohol, exercise, education, mean day BP, mean night BP, anti-hypertensive medications, DM, heart disease, the time between cognitive tests, and baseline cognitive scores.

Cognitive performance changes were calculated by subtracting baseline cognitive scores from follow-up scores of cognitive tests.

a Statistical significance was estimated after logarithmic transformation. BP, blood pressure; DM, diabetes mellitus; SBP, systolic blood pressure; SD, standard deviation. Bold values indicate *P* value <0.05.

We examined whether the relation between night systolic BPV and decline in cognitive function was mediated by GM atrophy (Supplementary Table 5). There was no significant evidence of mediation by GM volume atrophy in the relationship between night systolic BPV and cognitive decline in visual delayed recall memory (Sobel's test = 0.437, *P* = 0.662) or verbal fluency for category (Sobel's test = 0.432, *P* = 0.666).

## Discussion

We found that higher systolic BPV during the night was associated with the greater decline in brain volume and cognitive function over a mean follow-up of 4.3 years in a Korean population. Increased night BPV was associated with total brain volume atrophy, independent of average BP and the use of antihypertensive drugs. High variability of night SBP was associated with GM volume atrophy, especially temporal GM atrophy. Furthermore, the increase in night systolic BPV was associated with a greater decline in visual delayed recall memory and verbal fluency for category.

In our study, total brain volume atrophy and cognitive decline were associated with increased night BPV, but not with night mean BP. It is well-known that high BP contributes to cognitive impairments and dementia (3, 28). However, despite excellent BP control in a *post-hoc* analysis of the SPRINT MIND trial, higher BPV was associated with an increased risk of dementia (29), suggesting that increased BPV might be an independent factor associated with brain damage. Recent meta-analyses have demonstrated that increased BPV was associated with cerebral small vessel disease progression (10, 30), which is a major cause of cognitive decline (31–33). BPV also increased with age and mean BP level in our study. Therefore, high night BPV might be an epiphenomenon accompanied by the coexistence of various comorbidities or frailty in older adults. Increased BPV was associated with a greater risk of frailty (34), showing a significant correlation with cognitive decline, particularly in elderly hypertensive individuals (35–38).

Temporal GM was the area most affected by high night systolic BPV in our study. The relationship between night systolic BPV and temporal GM was driven most highly by atrophy of the left entorhinal cortex and right fusiform gyrus. To the best of our knowledge, this is the first study to identify the regional area of the brain most affected by night systolic and diastolic BPV. The entorhinal cortex is located in the medial temporal lobe, which functions as a widespread network hub for memory, navigation, and the perception of time (39). The fusiform gyrus is the largest macro-anatomical structure within the ventral temporal cortex and is considered a key structure for functionally specialized computations of high-level vision, such as face perception, object recognition, reading, and visual processing of letters and words (40). However, GM atrophy did not mediate the relationship between increased night systolic BPV and impaired visual delayed recall memory or verbal categorical fluency in our study. It remains to be seen whether brain regional connectivity or microstructural changes are involved in this relationship. More long-term follow-up studies are warranted to clarify the relevant mechanisms. It is unclear why the increased variation in night SBP was associated with slower decline in verbal recognition memory.

High night diastolic BPV was significantly related to WM atrophy, while the increase in night systolic BPV was associated with GM volume reduction in our study. It remains a matter of debate whether the variability of SBP or DBP contributes separately to GM and WM atrophy. Several studies have reported that DBP was strongly associated with WM hyperintensities (41, 42). It has been suggested that large artery stiffness leads to elevated SBP and pulse pressure, whereas DBP is reflecting peripheral vascular resistance (43). Brain pathology studies have demonstrated venous collagenosis in periventricular WM lesions (44). Therefore, it can be inferred that WM atrophy or lesion loads might be susceptible to changes in peripheral vascular resistance associated with DBP.

The exact mechanism by which increased night BPV, rather than daytime BPV, showed a significant correlation with cognitive decline and brain atrophy remains unknown. Daytime BPV might be directly affected by physical activities or emotional stress that occur during the day, whereas nocturnal BP monitoring could be less influenced by external stimuli. Increased night BPV might better reflect pathophysiological conditions such as baroreflex dysfunction, arterial stiffness neurohormonal activation, or sleep apnea than increased daytime BPV (45). Sleep is another possible key mechanism linked to fluctuations in nighttime BP. Sleep deprivation or fragmentation has been shown to be associated with increased night BPV through sympathetic neuronal activation (46, 47). Poor sleep quality has been linked to reduced brain volume and cognitive deficits (48, 49). However, in our study, there was no association between night BPV and sleep duration or snoring, and the significance of the results was not changed after adjustment for sleep-related indicators. The comprehensive association of several factors might account for the mechanisms associated with negative effects of high night BPV on cerebral vessels, brain structure, and function. Additional longitudinal studies are needed to determine whether dementia occurs more frequently in individuals with increased night BPV and if selective reduction of night BPV in this population could improve outcomes associated with brain health.

Our study has several strengths. First, our study is a 4-year, longitudinal, population-based study with a large sample size and brain MRI imaging data. Second, this study is the first to follow changes in detailed cognitive function tests in conjunction with brain imaging in relation to night BPV. Third, we examined sleep-related indicators as factors that could be associated with an increase in night BPV, although they were based on surveys. However, some limitations of this study should be noted. First, WM hyperintensities or diffusion MRI-based estimates of white matter microstructure were not measured in this study. These indicators of vascular brain injuries might be more sensitive to night BPV than brain volume loss, and further studies will be needed. Second, we did not address any associated neurohormonal changes during nighttime, which made it difficult to elucidate the mechanisms associated with increased night BPV. Third, there is a limit on the determination of the causal relationship between brain structural changes associated with high night BPV and cognitive decline.

To summarize, our study showed that increased nighttime BPV, rather than night mean BP, was associated with total brain volume atrophy and cognitive decline. High night systolic BPV was associated with temporal GM atrophy and impaired visual memory and verbal fluency. Increased nighttime BPV could be an independent predictor for rapid brain aging in a middle-aged population.

## Data availability statement

The data analyzed in this study was obtained from The Korea Centers for Disease Control and Prevention (KCDC), the following licenses/restrictions apply: The datasets can be provided after review and evaluation of research plan by the KCDC. Requests to access these datasets should be directed to the KCDC, http://www.cdc.go.kr/CDC/eng/main.jsp.

## Ethics statement

The study protocol was reviewed and approved by Institutional Review Board of Korea University Ansan Hospital. The participants provided written informed consent to participate in the cohort study.

## Author contributions

JY contributed to the study design and data analysis and wrote the manuscript. RK contributed to the MRI data processing and wrote the manuscript. SP and DL contributed to the literature search and data analysis. HC conducted the statistical analysis. NamK, HY, JS, SeK, SiK, KC, and SB reviewed and critiqued the statistical and MRI analyses and provided feedback on the manuscript text. CS and NanK contributed to data collection, obtained funding, and led the study. All authors read and approved the final manuscript, contributed toward data analysis, drafted and revised the paper, and agreed to be accountable for all aspects of the work.

## Funding

This work was supported by funds (2011-E71004-00, 2012-E71005-00, 2013-E71005-00, 2014-E71003-00, 2015-P71001-00, 2016-E71003-00, 2017-E71001-00, and 2018-E71001-00) from the Korean Centers for Disease Control and Prevention, the Bio & Medical Technology Development Program of the National Research Foundation (NRF) funded by the Korean government (MSIT) (2019M3E5D3073102, 2019R1H1A2039682, and 2020R1F1A1074265), a Korea University grant (K1824431 and K1810951), Ansan-Si hidden champion fostering and supporting project funded by Ansan city, and computational resources provided by the University of Iowa, Iowa City, Iowa.

## Conflict of interest

The authors declare that the research was conducted in the absence of any commercial or financial relationships that could be construed as a potential conflict of interest.

## Publisher's note

All claims expressed in this article are solely those of the authors and do not necessarily represent those of their affiliated organizations, or those of the publisher, the editors and the reviewers. Any product that may be evaluated in this article, or claim that may be made by its manufacturer, is not guaranteed or endorsed by the publisher.

## References

[1] Nagai M Hoshide S Ishikawa J Shimada K Kario K. Ambulatory blood pressure as an independent determinant of brain atrophy and cognitive function in elderly hypertension. J Hypertens. (2008) 26:1636–41. 10.1097/HJH.0b013e3283018333 18622243

[2] Papademetriou V. Hypertension and cognitive function. Blood pressure regulation and cognitive function: a review of the literature. Geriatrics. (2005) 60:20–4. 15700945

[3] Levine DA Springer MV Brodtmann A. Blood pressure and vascular cognitive impairment. Stroke. (2022) 53:1104–13. 10.1161/strokeaha.121.036140 35264009 PMC9141568

[4] Yano Y Inokuchi T Hoshide S Kanemaru Y Shimada K Kario K. Association of poor physical function and cognitive dysfunction with high nocturnal blood pressure level in treated elderly hypertensive patients. Am J Hypertens. (2011) 24:285–91. 10.1038/ajh.2010.224 21088668

[5] Yano Y Butler KR Hall ME Schwartz GL Knopman DS Lirette ST. Associations of nocturnal blood pressure with cognition by self-identified race in middle-aged and older adults: the GENOA (genetic epidemiology network of arteriopathy) study. J Am Heart Assoc. (2017) 6:e007022. 10.1161/jaha.117.007022 29079569 PMC5721781

[6] Ma JF Sun JL Zhao J Wei X Wang BS Fu Y. Relationship between nocturnal blood pressure variation and silent cerebral infarction in Chinese hypertensive patients. J Neurol Sci. (2010) 294:67–9. 10.1016/j.jns.2010.04.002 20439107

[7] Kario K Pickering TG Matsuo T Hoshide S Schwartz JE Shimada K. Stroke prognosis and abnormal nocturnal blood pressure falls in older hypertensives. Hypertension. (2001) 38:852–7. 10.1161/hy1001.092640 11641298

[8] Ma Y Wolters FJ Chibnik LB Licher S Ikram MA Hofman A. Variation in blood pressure and long-term risk of dementia: A population-based cohort study. PLoS Med. (2019) 16:e1002933. 10.1371/journal.pmed.1002933 31714941 PMC6850672

[9] Rothwell PM Howard SC Dolan E O'Brien E Dobson JE Dahlöf B. Prognostic significance of visit-to-visit variability, maximum systolic blood pressure, and episodic hypertension. Lancet. (2010) 375:895–905. 10.1016/s0140-6736(10)60308-x 20226988

[10] Ma Y Song A Viswanathan A Blacker D Vernooij MW Hofman A. Blood pressure variability and cerebral small vessel disease: a systematic review and meta-analysis of population-based cohorts. Stroke. (2020) 51:82–9. 10.1161/strokeaha.119.026739 31771460 PMC7050788

[11] Zhou TL Kroon AA van Sloten TT van Boxtel MPJ Verhey FRJ Schram MT. Greater blood pressure variability is associated with lower cognitive performance. Hypertension. (2019) 73:803–11. 10.1161/hypertensionaha.118.12305 30739535

[12] Yoo JE Shin DW Han K Kim D Lee SP Jeong SM. Blood pressure variability and the risk of dementia: a nationwide cohort study. Hypertension. (2020) 75:982–90. 10.1161/hypertensionaha.119.14033 32148122

[13] Qin B Viera AJ Muntner P Plassman BL Edwards LJ Adair LS. Visit-to-visit variability in blood pressure is related to late-life cognitive decline. Hypertension. (2016) 68:106–13. 10.1161/hypertensionaha.116.07494 27217401 PMC4900904

[14] Frattola A Parati G Cuspidi C Albini F Mancia G. Prognostic value of 24-h blood pressure variability. J Hypertens. (1993) 11:1133–7. 10.1097/00004872-199310000-00019 8258679

[15] Sander D Kukla C Klingelhöfer J Winbeck K Conrad B. Relationship between circadian blood pressure patterns and progression of early carotid atherosclerosis: A 3-year follow-up study. Circulation. (2000) 102:1536–41. 10.1161/01.cir.102.13.1536 11004145

[16] Goldstein IB Bartzokis G Guthrie D Shapiro D. Ambulatory blood pressure and the brain: a 5-year follow-up. Neurology. (2005) 64:1846–52. 10.1212/01.Wnl.0000164712.24389.Bb 15955932

[17] Yang S Yuan J Qin W Yang L Fan H Li Y. Twenty-four-hour ambulatory blood pressure variability is associated with total magnetic resonance imaging burden of cerebral small-vessel disease. Clin Interv Aging. (2018) 13:1419–27. 10.2147/cia.S171261 30127599 PMC6089119

[18] Goldstein IB Bartzokis G Guthrie D Shapiro D. Ambulatory blood pressure and brain atrophy in the healthy elderly. Neurology. (2002) 59:713–9. 10.1212/wnl.59.5.713 12221162

[19] Nakanishi K Jin Z Homma S Elkind MSV Rundek T Schwartz JE. Night-time systolic blood pressure and subclinical cerebrovascular disease: the cardiovascular abnormalities and brain lesions (CABL) study. Eur Heart J Cardiovasc Imag. (2019) 20:765–71. 10.1093/ehjci/jey221 30649236 PMC6587117

[20] Kim RE Yun CH Thomas RJ Oh JH Johnson HJ Kim S. Lifestyle-dependent brain change: a longitudinal cohort MRI study. Neurobiol Aging. (2018) 69:48–57. 10.1016/j.neurobiolaging.2018.04.017 29852410

[21] Kim RE Lourens S Long JD Paulsen JS Johnson HJ. Preliminary analysis using multi-atlas labeling algorithms for tracing longitudinal change. Front Neurosci. (2015) 9:242. 10.3389/fnins.2015.00242 26236182 PMC4500912

[22] Young Kim E Johnson HJ. Robust multi-site MR data processing: iterative optimization of bias correction, tissue classification, and registration. Front Neuroinform. (2013) 7:29. 10.3389/fninf.2013.00029 24302911 PMC3831347

[23] Ghayoor A Vaidya JG Johnson HJ. Robust automated constellation-based landmark detection in human brain imaging. Neuroimage. (2018) 170:471–81. 10.1016/j.neuroimage.2017.04.012 28392490 PMC5630513

[24] Wang H Yushkevich PA. Multi-atlas segmentation with joint label fusion and corrective learning-an open source implementation. Front Neuroinform. (2013) 7:27. 10.3389/fninf.2013.00027 24319427 PMC3837555

[25] Kim H Au R Thomas RJ Yun CH Lee SK Han C. Cognitive performance norms from the Korean genome and epidemiology study (KoGES). Int Psychogeriatr. (2017) 29:1909–24. 10.1017/s1041610217000990 28703093

[26] Genuth S Alberti KG Bennett P Buse J Defronzo R Kahn R. Follow-up report on the diagnosis of diabetes mellitus. Diabetes Care. (2003) 26:3160–7. 10.2337/diacare.26.11.3160 14578255

[27] Kim J Pack A Maislin G Lee SK Kim SH Shin C. Prospective observation on the association of snoring with subclinical changes in carotid atherosclerosis over four years. Sleep Med. (2014) 15:769–75. 10.1016/j.sleep.2014.03.009 24841110 PMC4127330

[28] Hughes D Judge C Murphy R Loughlin E Costello M Whiteley W. Association of blood pressure lowering with incident dementia or cognitive impairment: a systematic review and meta-analysis. Jama. (2020) 323:1934–44. 10.1001/jama.2020.4249 32427305 PMC7237983

[29] de Havenon A Anadani M Prabhakaran S Wong KH Yaghi S Rost N. Increased blood pressure variability and the risk of probable dementia or mild cognitive impairment: a *post-hoc* analysis of the sprint mind trial. J Am Heart Assoc. (2021) 10:e022206. 10.1161/jaha.121.022206 34533059 PMC8649507

[30] Tully PJ Yano Y Launer LJ Kario K Nagai M Mooijaart SP. Association between blood pressure variability and cerebral small-vessel disease: a systematic review and meta-analysis. J Am Heart Assoc. (2020) 9:e013841. 10.1161/jaha.119.013841 31870233 PMC6988154

[31] Gyanwali B Vrooman H Venketasubramanian N Wong TY Cheng CY Chen C. Cerebral small vessel disease and enlarged perivascular spaces-data from memory clinic and population-based settings. Front Neurol. (2019) 10:669. 10.3389/fneur.2019.00669 31293506 PMC6603207

[32] Liu R Chen H Qin R Gu Y Chen X Zou J. The altered reconfiguration pattern of brain modular architecture regulates cognitive function in cerebral small vessel disease. Front Neurol. (2019) 10:324. 10.3389/fneur.2019.00324 31024423 PMC6461194

[33] Fan Y Xu Y Shen M Guo H Zhang Z. Total cerebral small vessel disease burden on mri correlates with cognitive impairment in outpatients with amnestic disorders. Front Neurol. (2021) 12:747115. 10.3389/fneur.2021.747115 34925212 PMC8675386

[34] Rouch L De Souto Barreto P Hanon O Vidal JS Amar J Andrieu S. Visit-to-visit blood pressure variability and incident frailty in older adults. J Gerontol A Biol Sci Med Sci. (2021) 76:1369–75. 10.1093/gerona/glab112 33844014

[35] Mone P Pansini A Calabrò F De Gennaro S Esposito M Rinaldi P. Global cognitive function correlates with P-wave dispersion in frail hypertensive older adults. J Clin Hypertens. (2022) 24:638–43. 10.1111/jch.14439 35229449 PMC9106080

[36] Mone P Gambardella J Lombardi A Pansini A De Gennaro S Leo AL. Correlation of physical and cognitive impairment in diabetic and hypertensive frail older adults. Cardiovasc Diabetol. (2022) 21:10. 10.1186/s12933-021-01442-z 35045834 PMC8772197

[37] Mone P Pansini A Frullone S de Donato A Buonincontri V De Blasiis P. Physical decline and cognitive impairment in frail hypertensive elders during COVID-19. Eur J Intern Med. (2022) 99:89–92. 10.1016/j.ejim.2022.03.012 35300886 PMC8919809

[38] Matsue Y Kamiya K Saito H Saito K Ogasahara Y Maekawa E. Prevalence and prognostic impact of the coexistence of multiple frailty domains in elderly patients with heart failure: the FRAGILE-HF cohort study. Eur J Heart Fail. (2020) 22:2112–9. 10.1002/ejhf.1926 32500539

[39] Tsao A Sugar J Lu L Wang C Knierim JJ Moser MB. Integrating time from experience in the lateral entorhinal cortex. Nature. (2018) 561:57–62. 10.1038/s41586-018-0459-6 30158699

[40] Weiner KS Zilles K. The anatomical and functional specialization of the fusiform gyrus. Neuropsychologia. (2016) 83:48–62. 10.1016/j.neuropsychologia.2015.06.033 26119921 PMC4714959

[41] Wartolowska KA Webb AJS. Midlife blood pressure is associated with the severity of white matter hyperintensities: analysis of the UK Biobank cohort study. Eur Heart J. (2021) 42:750–7. 10.1093/eurheartj/ehaa756 33238300 PMC7882359

[42] Marcus J Gardener H Rundek T Elkind MS Sacco RL Decarli C. Baseline and longitudinal increases in diastolic blood pressure are associated with greater white matter hyperintensity volume: the northern Manhattan study. Stroke. (2011) 42:2639–41. 10.1161/strokeaha.111.617571 21836088 PMC3189513

[43] Guo X Pantoni L Simoni M Bengtsson C Björkelund C Lissner L. Blood pressure components and changes in relation to white matter lesions: a 32-year prospective population study. Hypertension. (2009) 54:57–62. 10.1161/hypertensionaha.109.129700 19487586

[44] Moody DM Brown WR Challa VR Ghazi-Birry HS Reboussin DM. Cerebral microvascular alterations in aging, leukoaraiosis, and Alzheimer's disease. Ann N Y Acad Sci. (1997) 826:103–16. 10.1111/j.1749-6632.1997.tb48464.x 9329684

[45] Mancia G Grassi G. Mechanisms and clinical implications of blood pressure variability. J Cardiovasc Pharmacol. (2000) 35:S15–9. 10.1097/00005344-200000004-00003 11346215

[46] Somers VK Dyken ME Mark AL Abboud FM. Sympathetic-nerve activity during sleep in normal subjects. N Engl J Med. (1993) 328:303–7. 10.1056/nejm199302043280502 8419815

[47] Dettoni JL Consolim-Colombo FM Drager LF Rubira MC Souza SB Irigoyen MC. Cardiovascular effects of partial sleep deprivation in healthy volunteers. J Appl Physiol. (2012) 113:232–6. 10.1152/japplphysiol.01604.2011 22539169

[48] Sexton CE Storsve AB Walhovd KB Johansen-Berg H Fjell AM. Poor sleep quality is associated with increased cortical atrophy in community-dwelling adults. Neurology. (2014) 83:967–73. 10.1212/wnl.0000000000000774 25186857 PMC4162301

[49] Benitez A Gunstad J. Poor sleep quality diminishes cognitive functioning independent of depression and anxiety in healthy young adults. Clin Neuropsychol. (2012) 26:214–23. 10.1080/13854046.2012.658439 22335237

