# Supplementary material for: Night blood pressure variability, brain atrophy, and cognitive decline
Source: Front Neurol. 2022 Sep 1;13:963648. doi: 10.3389/fneur.2022.963648 (PMC9474888; doi:10.3389/fneur.2022.963648)
Supplement: Supplementary file 1 [file Data_Sheet_1.docx]

Supplementary Material

**Supplementary Table 1.** Multivariate Regression Analyses for the Associations between Night BPV and Clinical Factors.

|  | SD of night SBP^a^ | | |  | SD of night DBP^b^ | | |
| --- | --- | --- | --- | --- | --- | --- | --- |
|  | β Estimate | SE | *p* |  | β Estimate | SE | *p* |
| Age, years | 0.097 | 0.019 | **<0.001** |  | 0.082 | 0.015 | **<0.001** |
| Sex | -0.173 | 0.280 | 0.536 |  | 0.291 | 0.224 | 0.193 |
| BMI | 0.104 | 0.042 | **0.013** |  | 0.177 | 0.032 | **<0.001** |
| Smoking | 0.669 | 0.419 | 0.111 |  | 0.292 | 0.320 | 0.363 |
| Alcohol | 0.168 | 0.268 | 0.533 |  | -0.003 | 0.206 | 0.990 |
| Exercise | -0.229 | 0.239 | 0.339 |  | -0.144 | 0.183 | 0.432 |
| Education | 0.188 | 0.362 | 0.602 |  | 0.400 | 0.277 | 0.148 |
| DM | -0.072 | 0.257 | 0.779 |  | 0.380 | 0.197 | 0.053 |
| Heart disease | 0.080 | 0.447 | 0.857 |  | 0.146 | 0.342 | 0.669 |
| Average day BP, mmHg | 0.058 | 0.014 | **<0.001** |  | 0.104 | 0.014 | **<0.001** |
| Average night BP, mmHg | 0.041 | 0.013 | **0.003** |  | -0.041 | 0.014 | **0.003** |
| Sleep duration, h | -0.161 | 0.094 | 0.088 |  | -0.071 | 0.072 | 0.321 |
| Snoring | 0.533 | 0.279 | 0.056 |  | 0.351 | 0.214 | 0.101 |

The regression models included SD of night BP as the dependent variable; age, sex, BMI, smoking, alcohol, exercise, education, DM, heart disease, average of day and night BP, sleep duration, and snoring were included as the independent variables. ^a^The average SBP during the day and that during the night were included in the regression model. ^b^The average DBP during the day and that during the night were included in the regression model. BMI, body mass index; BP, blood pressure; BPV, blood pressure variability; DBP, diastolic blood pressure; DM, diabetes mellitus; SBP, systolic blood pressure; SD, standard deviation.

**Supplementary Table 2.** Linear regression analyses between night BPV and regional volume changes in temporal GM

|  | **SD of night SBP** | | |  | **SD of night DBP** | | |
| --- | --- | --- | --- | --- | --- | --- | --- |
| **Δ µl (n=1398)** | **β Estimate** | **SE** | ***p*** |  | **β Estimate** | **SE** | ***p*** |
| **Temporal GM, Left** |  |  |  |  |  |  |  |
| Δ entorhinal | -2.003 | 0.779 | **0.010** |  | -1.857 | 1.011 | 0.067 |
| Δ fusiform | -1.589 | 3.220 | 0.622 |  | -3.876 | 4.174 | 0.353 |
| Δ inferior temporal | -2.069 | 4.129 | 0.616 |  | 4.275 | 5.352 | 0.425 |
| Δ middle temporal | -5.858 | 4.481 | 0.191 |  | -0.886 | 5.816 | 0.879 |
| Δ planum temporale | -0.811 | 0.888 | 0.361 |  | 0.397 | 1.150 | 0.730 |
| Δ superior temporal gyrus part A | -0.106 | 2.193 | 0.961 |  | 0.187 | 2.843 | 0.948 |
| Δ superior temporal gyrys part B | -0.283 | 0.550 | 0.607 |  | 0.186 | 0.712 | 0.794 |
| Δ temporal pole | -1.539 | 2.883 | 0.594 |  | -1.975 | 3.739 | 0.597 |
| Δ transverse temporal | -0.283 | 0.913 | 0.757 |  | -0.971 | 1.183 | 0.412 |
| Δ parahippocampal | -0.037 | 0.822 | 0.964 |  | 0.273 | 1.066 | 0.798 |
| **Temporal GM, Right** |  |  |  |  |  |  |  |
| Δ entorhinal | -1.506 | 0.865 | 0.082 |  | -1.223 | 1.122 | 0.276 |
| Δ fusiform | -5.576 | 2.701 | **0.039** |  | -8.331 | 3.501 | **0.018** |
| Δ inferior temporal | -6.724 | 3.718 | 0.071 |  | -2.587 | 4.820 | 0.592 |
| Δ middle temporal | -4.348 | 4.301 | 0.312 |  | 2.053 | 5.580 | 0.713 |
| Δ planum temporale | -0.322 | 0.743 | 0.665 |  | 0.577 | 0.964 | 0.549 |
| Δ superior temporal gyrus part A | -3.311 | 2.163 | 0.126 |  | -0.765 | 2.804 | 0.785 |
| Δ superior temporal gyrus part B | -0.298 | 0.515 | 0.563 |  | 0.054 | 0.668 | 0.936 |
| Δ temporal pole | -1.140 | 2.786 | 0.683 |  | -3.101 | 3.605 | 0.390 |
| Δ transverse temporal | -0.524 | 0.517 | 0.310 |  | -0.624 | 0.670 | 0.352 |
| Δ parahippocampal | -0.149 | 0.855 | 0.862 |  | -0.036 | 1.109 | 0.974 |

All models were adjusted for baseline intracranial volume, age, sex, smoking, alcohol, exercise, education, mean day BP, mean night BP, anti-hypertensive medications, DM, heart disease, time between MRI scans, and baseline brain volume. Regional brain volume changes were calculated by subtracting baseline regional brain volumes from follow-up volumes. BPV, blood pressure variability; DBP, diastolic blood pressure; GM, gray matter; MRI, magnetic resonance imaging; SBP, systolic blood pressure.

**Supplementary Table 3.** Linear regression analyses between night BPV and regional volume changes in WM

|  | **SD of night SBP** | | |  | **SD of night DBP** | | |
| --- | --- | --- | --- | --- | --- | --- | --- |
| **Δ µl (n=1398)** | **β Estimate** | **SE** | ***p*** |  | **β Estimate** | **SE** | ***p*** |
| **WM, Left** |  |  |  |  |  |  |  |
| Δ precentral | -4.590 | 4.613 | 0.320 |  | -9.501 | 5.974 | 0.112 |
| Δ paracentral | -1.482 | 1.310 | 0.258 |  | -3.816 | 1.695 | **0.025** |
| Δ rostral middle frontal | 0.888 | 4.745 | 0.852 |  | -3.449 | 6.155 | 0.575 |
| Δ superior frontal | -11.608 | 6.617 | 0.080 |  | -19.433 | 8.569 | **0.024** |
| Δ precuneus | -7.359 | 3.619 | **0.042** |  | -10.008 | 4.692 | **0.033** |
| Δ transverse temporal | -0.245 | 0.162 | 0.130 |  | -0.466 | 0.210 | **0.027** |
| Δ lingual | -5.304 | 1.992 | **0.008** |  | -6.246 | 2.585 | **0.016** |
| Δ cuneus | -0.958 | 0.568 | 0.092 |  | -1.164 | 0.736 | 0.114 |
| **WM, Right** |  |  |  |  |  |  |  |
| Δ precentral | -7.163 | 5.213 | 0.170 |  | -13.518 | 6.749 | **0.045** |
| Δ paracentral | -1.585 | 1.785 | 0.375 |  | -2.812 | 2.313 | 0.224 |
| Δ rostral middle frontal | -4.458 | 5.600 | 0.426 |  | -14.375 | 7.257 | **0.048** |
| Δ superior frontal | -9.765 | 6.205 | 0.116 |  | -15.866 | 8.041 | **0.049** |
| Δ precuneus | -6.419 | 4.658 | 0.168 |  | -11.174 | 6.033 | 0.064 |
| Δ transverse temporal | -0.094 | 0.216 | 0.663 |  | -0.224 | 0.280 | 0.424 |
| Δ lingual | -2.073 | 1.809 | 0.252 |  | -4.981 | 2.342 | **0.034** |
| Δ cuneus | -1.393 | 0.591 | **0.019** |  | -1.671 | 0.767 | **0.029** |

All models were adjusted for baseline intracranial volume, age, sex, smoking, alcohol, exercise, education, mean day BP, mean night BP, anti-hypertensive medications, DM, heart disease, time between MRI scans, and baseline brain volume. Regional brain volume changes were calculated by subtracting baseline regional brain volumes from follow-up volumes. BPV, blood pressure variability; DBP, diastolic blood pressure; GM, gray matter; MRI, magnetic resonance imaging; SBP, systolic blood pressure.

**Supplementary Table 4.** Linear regression analyses between night mean BP and cognitive performance changes

|  | Night mean SBP | | |  | Night mean DBP | | |
| --- | --- | --- | --- | --- | --- | --- | --- |
|  | Estimate | SE | *P* |  | Estimate | SE | *P* |
| Story Recall Test-Immediate Recall | -0.005 | 0.011 | 0.637 |  | -0.019 | 0.015 | 0.192 |
| Story Recall Test-Delayed recall | -0.003 | 0.011 | 0.813 |  | -0.009 | 0.015 | 0.524 |
| Story Recall Test-Recognition^a^ | 0.001 | 0.001 | 0.095 |  | 0.001 | 0.001 | 0.405 |
| Visual Reproductions-Immediate Recall | -0.004 | 0.007 | 0.494 |  | -0.007 | 0.009 | 0.438 |
| Visual Reproductions-Delayed Recall | -0.009 | 0.007 | 0.190 |  | -0.010 | 0.009 | 0.286 |
| Visual Reproductions-Recognition | 0.004 | 0.003 | 0.231 |  | 0.002 | 0.004 | 0.649 |
| Verbal fluency-Phonemic | -0.014 | 0.022 | 0.506 |  | 0.011 | 0.029 | 0.707 |
| Verbal fluency-Category | 0.011 | 0.010 | 0.284 |  | 0.016 | 0.013 | 0.232 |
| Digit symbol-Coding | -0.010 | 0.020 | 0.625 |  | 0.002 | 0.027 | 0.936 |
| Digit symbol-Incidental learning^a^ | 0.000 | 0.002 | 0.831 |  | -0.002 | 0.003 | 0.444 |
| Digit symbol-Free recall | 0.003 | 0.004 | 0.409 |  | 0.006 | 0.005 | 0.288 |
| Trails A-Time^a^ | 0.001 | 0.001 | 0.458 |  | 0.000 | 0.001 | 0.841 |
| Stroop-Color reading | 0.063 | 0.067 | 0.345 |  | 0.067 | 0.089 | 0.450 |
| Stroop-Word reading | 0.012 | 0.022 | 0.607 |  | 0.020 | 0.030 | 0.492 |

All models were adjusted for baseline intracranial volume, age, sex, smoking, alcohol, exercise, education, mean day BP, anti-hypertensive medications, DM, heart disease, time between cognitive tests, and baseline cognitive scores.

Cognitive performance changes were calculated by subtracting baseline cognitive scores from follow-up scores of cognitive tests.

^a^Statistical significance was estimated after logarithmic transformation.

BP, blood pressure; DM, diabetes mellitus; DBP, diastolic blood pressure; SBP, systolic blood pressure.

**Supplementary Table 5. Mediation effects of GM atrophy in the association between night systolic BPV and cognitive decline**

|  | Effect | |  | Effect | |  | Total  effect |  | Direct  effect |  | Indirect  effect | Sobel Statistics | Sobel  P value |
| --- | --- | --- | --- | --- | --- | --- | --- | --- | --- | --- | --- | --- | --- |
| Cognitive score changes | of IV on M | |  | of M on DV | |  |  |  |  |  |  |  |  |
| (=DV) | a^a^ | SE |  | b^b^ | SE |  | c^c^ |  | d^d^ |  | a x b^e^ |  |  |
| Story Recall Test-Immediate Recall | -0.142 | 0.077 |  | -0.011 | 0.020 |  | -0.122 |  | -0.123 |  | 0.002 | 0.541 | 0.588 |
| Story Recall Test-Delayed recall | -0.142 | 0.077 |  | -0.005 | 0.020 |  | -0.134 |  | -0.134 |  | 0.001 | 0.236 | 0.813 |
| Story Recall Test-Recognition^f^ | -0.142 | 0.077 |  | -0.000 | 0.000 |  | -0.003 |  | -0.003 |  | 0.000 | 0.578 | 0.563 |
| Visual Reproductions-Immediate Recall | -0.142 | 0.077 |  | 0.002 | 0.006 |  | -0.051 |  | -0.051 |  | -0.000 | -0.251 | 0.802 |
| Visual Reproductions-Delayed Recall | -0.142 | 0.077 |  | -0.003 | 0.007 |  | -0.045 |  | -0.045 |  | 0.000 | 0.437 | 0.662 |
| Visual Reproductions-Recognition | -0.142 | 0.077 |  | -0.004 | 0.002 |  | -0.008 |  | -0.008 |  | 0.001 | 1.295 | 0.195 |
| Verbal fluency-Phonemic | -0.142 | 0.077 |  | -0.036 | 0.020 |  | -0.109 |  | -0.115 |  | 0.005 | 1.274 | 0.203 |
| Verbal fluency-Category | -0.142 | 0.077 |  | -0.004 | 0.008 |  | -0.001 |  | -0.001 |  | 0.001 | 0.432 | 0.666 |
| Digit symbol-Coding | -0.142 | 0.077 |  | -0.037 | 0.037 |  | -0.486 |  | -0.492 |  | 0.005 | 0.871 | 0.384 |
| Digit symbol-Incidental learning^f^ | -0.142 | 0.077 |  | -0.002 | 0.002 |  | -0.008 |  | -0.009 |  | 0.000 | 1.088 | 0.277 |
| Digit symbol-Free recall | -0.142 | 0.077 |  | 0.001 | 0.003 |  | 0.001 |  | 0.001 |  | -0.000 | -0.295 | 0.768 |
| Trails A-Time^f^ | -0.142 | 0.077 |  | 0.001 | 0.001 |  | 0.008 |  | 0.008 |  | -0.000 | -0.671 | 0.502 |
| Stroop-Color reading | -0.142 | 0.077 |  | -0.013 | 0.043 |  | -0.434 |  | -0.436 |  | 0.002 | 0.304 | 0.761 |
| Stroop-Word reading | -0.142 | 0.077 |  | -0.032 | 0.031 |  | -0.264 |  | -0.266 |  | 0.005 | 0.899 | 0.369 |

^a^a, Effect of night systolic BPV on GM atrophy. ^b^b, Effect of GM atrophy on cognitive decline. ^c^c, Total effect of night systolic BPV on cognitive decline. ^d^d, Direct effect of night systolic BPV on cognitive decline. ^e^a x b, Indirect effect of night systolic BPV on cognitive decline via GM atrophy.

Cognitive score changes were calculated by subtracting baseline cognitive scores from follow-up scores of cognitive tests.

^f^Statistical significance was estimated after logarithmic transformation.

BPV, blood pressure variability; DV, dependent variable (=cognitive score changes); GM, gray matter; IV, independent variable (=night systolic BPV); M, mediator (=GM atrophy); SE, standard error
